# Integrated analysis of lncRNA and mRNA expression profiles in cutaneous leishmaniasis lesions caused by *Leishmania tropica*

**DOI:** 10.3389/fcimb.2024.1416925

**Published:** 2024-11-21

**Authors:** Shima Hadifar, Nasrin Masoudzadeh, Björn Andersson, Hossein Heydari, Vahid Mashayekhi Goyonlo, Mohammadali Kerachian, Josefine Persson, Hasan Rahimi-Tamandegani, Reza Erfanian Salim, Sima Rafati, Ali M. Harandi

**Affiliations:** ^1^ Department of Immunotherapy and Leishmania Vaccine Research, Pasteur Institute of Iran, Tehran, Iran; ^2^ Bioinformatics Core Facility, Sahlgrenska Academy, University of Gothenburg, Gothenburg, Sweden; ^3^ Cutaneous Leishmaniasis Research Center, Mashhad University of Medical Sciences, Mashhad, Iran; ^4^ Department of Microbiology and Immunology, Institute of Biomedicine, Sahlgrenska Academy, University of Gothenburg, Gothenburg, Sweden; ^5^ Medical Biotechnology Department, Pasteur Institute of Iran, Tehran, Iran; ^6^ Noor Eye Hospital, Tehran, Iran

**Keywords:** cutaneous leishmaniasis, *Leishmania tropica*, lncRNAs, WGCNA, transcriptomics, co-expression network

## Abstract

**Background:**

Cutaneous leishmaniasis (CL), caused by *Leishmania* (*L.*) species, remains a neglected tropical disease in many developing countries. We and others have shown that different *Leishmania* species can alter the gene expression profile of human host cells. Long non-coding RNAs (lncRNAs) have been found to play a role in the pathogenesis of leishmaniasis through dysregulation of transcriptome signatures. Understanding the regulatory roles of lncRNAs in the biological networks involved in leishmaniasis can improve our understanding of the disease.

**Methods:**

Herein, we used our previous RNA sequencing data (GSE216638) to investigate the profile of lncRNAs in the skin lesions of *L. tropica*-infected patients. We employed the weighted gene correlation network analysis (WGCNA) algorithm to establish co-expression networks of shared genes between CL patients and infer the potential role of lncRNAs in CL patients. We identified hub genes and trans- and cis-acting lncRNAs, and carried out functional enrichment analysis on a key co-expressed module related to *L. tropica*-infected patients.

**Results:**

We found substantial involvement of lncRNAs in the CL patient dataset. Using the WGCNA method, we classified all included genes into seven modules, with a module (turquoise) being significantly correlated with the studied clinical traits and identified as the key module. This module was mainly involved in the “interferon gamma signaling” and “cytokine signaling” pathways. We highlighted several lncRNAs and their co-expressed mRNA pairs, like SIRPG-AS1, IL21R-AS1, IL24, and TLDC2, as hub genes of the key module. Quantitative RT-PCR validated the expression of several genes in the lesions of an independent cohort of *L. tropica*-infected patients.

**Conclusions:**

These findings enhance our understanding of the human skin response to *L. tropica* infection. Furthermore, the hub genes identified in this study are worthy of further evaluation as potential targets in the development of more effective treatments and preventive measures for CL caused by *L. tropica*.

## 1 Introduction

Leishmaniasis, a vector-borne disease caused by protozoan parasites of the genus *Leishmania*, can lead to a spectrum of severe immunopathologies ranging from self-healing cutaneous leishmaniasis (CL) to fatal visceral leishmaniasis (VL). The severity of the disease depends on the parasite and vector species as well as host traits. CL is the most common form, with an estimated 0.6 to 1 million new cases annually (Ruiz-Postigo et al., 2021). Combating the disease is challenging due to the complex and interactive factors involved in the transmission cycle of the parasite in human hosts, vectors, and reservoirs (Bañuls et al., 2011). A holistic approach, such as transcriptome analysis, may offer valuable insights into understanding the complexity of CL in different human populations (Masoudzadeh et al., 2020a). Although transcriptome profiles of CL patients infected by species such as *L. braziliensis* (Amorim et al., 2019), *L. amazonensis* (Christensen et al., 2019), and *L. tropica* (Masoudzadeh et al., 2020b) have been reported, the precise molecular mechanism involved in the regulatory network of human CL has not yet been fully elucidated.

Long non-coding RNAs (lncRNAs) are emerging as a new regulator of mammalian immune responses during host–pathogen interactions (Zhang et al., 2019; Statello et al., 2021). They are a heterogeneous class of non-coding RNA molecules with a length greater than 200 base pairs (Mattick et al., 2023), which can modulate gene expression by acting as decoys, guides, scaffolds, or sponges (Wang and Chang, 2011). The tissue-specific expression patterns of most lncRNA genes suggest that their expression is highly regulated during development and pathogenesis. Moreover, dysregulated lncRNA signatures have been found to be involved in the pathogenesis of various diseases (DiStefano, 2018; Kopp and Mendell, 2018). More recent evidence has also suggested a potential role of lncRNAs in the development and progression of leishmaniasis caused by different *Leishmania* species (Fernandes et al., 2022; Maruyama et al., 2022; Almeida et al., 2023). However, the involvement of lncRNAs in the regulation of human skin response to CL caused by *L. tropica* has not been thoroughly investigated.

In the current study, a co-expression network of lncRNA and mRNA genes [retrieved from our RNA sequencing (RNA-seq) dataset] was constructed by the weighted gene correlation network analysis (WGCNA) algorithm (Langfelder and Horvath, 2008) to infer the potential role of lncRNAs in the skin lesions of CL caused by *L. tropica*. The WGCNA of integrated coding and non-coding gene data linked lncRNAs with unknown functions to biological processes through determining correlated lncRNA and mRNA pairs. Using quantitative real-time polymerase chain reaction (qRT-PCR), we validated several identified potential hub genes specifically associated with CL. These results unveil new insights into the integrated regulatory networks (lncRNAs–mRNAs) involved in the transcriptional alterations induced by *L. tropica* in human skin and can inform the identification of novel potential targets for effective intervention strategies.

## 2 Materials and methods

### 2.1 Data collection

We utilized our previously published RNA-seq data from CL patients infected with *L. tropica*. The data are available on the NCBI Gene Expression Omnibus (GEO) database under accession number GSE216638 (Masoudzadeh et al., 2020b). Briefly, skin biopsy samples were collected from 6 healthy volunteers and 16 *L. tropica*-infected CL patients dermatologically classified into two groups: 7 ulcerative CLs (UCLs) and 9 non-ulcerated CLs (NUCLs).

### 2.2 RNA−seq data analysis

RNA-seq data from UCL and NUCL patients were analyzed as described previously (Masoudzadeh et al., 2020b). To summarize, the clean reads (FASTQ) data were aligned to the human genome (hg19/GRCh38) using the Star tool (Dobin et al., 2013). Then, to determine the mRNA and lncRNA genes in the included RNA-seq data, human gene transfer format (GTF) files downloaded from Ensembl (http://asia.ensembl.org) were used to annotate genes in each sample by HTSeq (Anders et al., 2015). The R package DESeq2 (Love et al., 2014) was used to find the differentially expressed lncRNA gene list between patients and the healthy groups. The adjusted *p*-value <0.05 was considered statistically significant. Up- and downregulated differentially expressed genes (DEGs) were defined by the criteria of log2 |fold change (FC)| > 1.

To identify the shared and specific lncRNA and mRNA genes, a Venn diagram was generated using the VennDiagram (v.1.7.3) package in R (v 4.1.1, https://www.r-project.org/) (Team R and Team RC, 2018). To show significantly expressed lncRNA and mRNA genes, volcano plots were generated by ggplot2 (v 3.3.5) and ggrepel (v 0.9.1) packages.

### 2.3 Weighted gene correlation network analysis

WGCNA was used to explore network modules highly associated with our clinical traits. For WGCNA analysis, we screened mRNA and lncRNA gene lists by focusing only on statistically significant genes (adjusted *p*-value < 0.05). Subsequently, we identified the lists of significant genes common to both clinical forms of CL, UCLs and NUCLs, and visualized the results using a Venn diagram. The shared significantly expressed lncRNA and mRNA genes were prioritized for making a coding–non-coding gene co-expression network using the WGCNA R package (v1. 70-3) (Langfelder and Horvath, 2008). We integrated the lncRNA and mRNA data to find the correlations between them and subsequently detect hub lncRNA genes. The hclust function of WGCNA was used to identify outlier samples in the included data (22 samples). A signed weighted correlation matrix considering Pearson’s correlations between the selected genes across all the samples (22 samples) was computed using an optimal soft threshold of *β* = 15 (scale-free *R* ^2^ = 0.91). The disease-specific adjacency matrices were transformed into the topological overlap matrix (TOM). Then, the topological overlap dissimilarity matrix (1-TOM) value was computed to generate a hierarchical clustering tree. Finally, the WGCNA cutreeDynamic function was used to identify the consensus co-expressed gene network modules with a minimum module size of 25 and a deep Split of two. The mergeCutHeight parameter of modules was also set according to the dissimilarity of calculated module eigengenes (MEs), which is the first principal component of the principal component analysis of gene expression profile in a module.

#### 2.3.1 Identification of key modules, hub genes, and cis- and trans-interactions

Two subsequent analyses were conducted to identify the key module in CL patients. The correlation between the ME and UCL–NUCL (common genes between UCL and NUCL) as the clinical trait was calculated to find a module–trait association. Modules with an absolute correlation of >0.6 and *p* < 0.05 were selected for subsequent analysis. At the next level, to detect key modules (or significant modules), Pearson’s correlation of gene significance (GS) and module membership (MM) for each filtered module was calculated. MM defines the correlation between the gene expression profile and the ME. GS refers to the correlation between the gene expression profile and clinical traits. The module with the highest statistically significant absolute correlation value was determined as the key module. Following that, Cytoscape (v3. 9) was used to visualize the genes’ network of key modules (the threshold for connectivity weight was set to 0.3) (Kohl et al., 2011).

To screen out the DE mRNA hub genes, regarded as topologically and functionally the more significant genes than other genes in the key modules, we set a minimum threshold for these two parameters, genes with |GS | > 0.6 and MM > 0.6 plus having the highest value for degree in the genes’ network of the modules (intra-modular connectivity). In addition, we selected the hub DE lncRNAs in the key module based on the above criteria for selecting hub mRNAs and interacting with at least one of the selected hub mRNAs in the previous step. To visualize the selected hub genes, heatmap plots were constructed using the pheatmap R package (v1.0.12) (Kolde, 2012). Next, to provide more in-depth information on the molecular function of selected hub lncRNAs, subcellular localization was predicted using the sequence-based web tool of LightGBM_LncLoc (Lyu et al., 2023).

In order to explore the potential functions of the identified DE lncRNAs and regulatory relationships between lncRNAs and mRNAs in the key module, cis- and trans-targets of the DE lncRNAs were investigated through the positional relationship and expression correlation, respectively. Protein-coding genes that matched our co-expressed genes and positioned within a genomic window of 100 kb upstream and downstream of DE lncRNAs were used to investigate the probable cis-target of DE lncRNAs in the key module. Prediction of trans-target genes of lncRNA was based on the correlation value of co-expressed DE lncRNA–mRNA pairs (| ***r*** | > 0.95) in the key module. Furthermore, the interactions of hub lncRNAs with transcription factors (TFs) were predicted using the RNAInter database (Lin et al., 2020).

### 2.4 Gene ontology and pathway enrichment analysis

To explore the main biological processes driven by the key module with hub genes, gene ontology (GO) on the basis of biological process and Reactome pathway enrichment analysis (Fabregat et al., 2017) were performed. ClueGo plugins in Cytoscape were used to visualize the enrichment results in a network-based manner. The enrichment result with a *p*-value < 0.05 was considered statistically significant.

### 2.5 Sample collection

The CL patients were selected from the clinic at Mashhad Medical School in Mashhad, Iran, which is a highly endemic area for *L. tropica*. None of the patients had received anti-leishmanial treatment. Sample collection for diagnosis was done using a non-invasive tape disc-based sampling method as described elsewhere (Taslimi et al., 2017). All CL patients had active lesions for a maximum of 1 year and tested positive for *L. tropica* by specific PCR tests. Two PCR tests were conducted to determine the species of *Leishmania*: the first targeted the *Leishmania* kDNA minicircle and the second employed the ITS1 genes (Taslimi et al., 2017).

Skin biopsy samples from 28 participants, 18 CL patients and 10 healthy controls, were taken (**Supplementary Table S1**) and promptly stored at −80°C in RNA later (Qiagen GmbH, Hilden, Germany). The healthy skin samples were obtained from volunteers undergoing esthetical surgery at Tehran’s Noor Eye Hospital, who had no history of leishmaniasis or any other skin problems.

### 2.6 Quantitative real-time RT-PCR

To validate RNA-seq data, the expression of selected mRNA and lncRNA genes was assessed using the qRT-PCR method. Total RNA was extracted from skin biopsy samples using a total RNA extraction mini kit (Pars tous, Cat. No. A101231, Iran) according to the manufacturer’s instructions. For genomic DNA removal, RNA was treated with DNase I (Ambion, Cat. No. AM1906, USA) and then cDNA synthesis was performed by High-Capacity cDNA Reverse Transcription Kits (Applied Biosystems, Cat. No. 4368813, USA) following the manufacturer’s instructions. qRT-PCR was performed by the QuantiFast SYBR Green PCR Kit (Qiagen, Cat. No. 204056, GmbH, Hilden, Germany) in the ABI Real-time PCR Detection System (Applied Biosystems, CA, USA). The primer sequences are shown in **Supplementary Table S2**.

### 2.7 Statistical analysis

The ΔΔCT method was used to analyze relative gene expression, with human GAPDH serving as the endogenous control. To compare the differences between the two groups, we used the Mann–Whitney ***U*** test. A *p*-value of less than 0.05 was considered statistically significant. All analyses were performed using GraphPad Prism 8.0 (GraphPad Software Inc. CA, USA).

## 3 Results

### 3.1 Identification of differentially expressed lncRNAs and mRNAs

**Figure 1:**
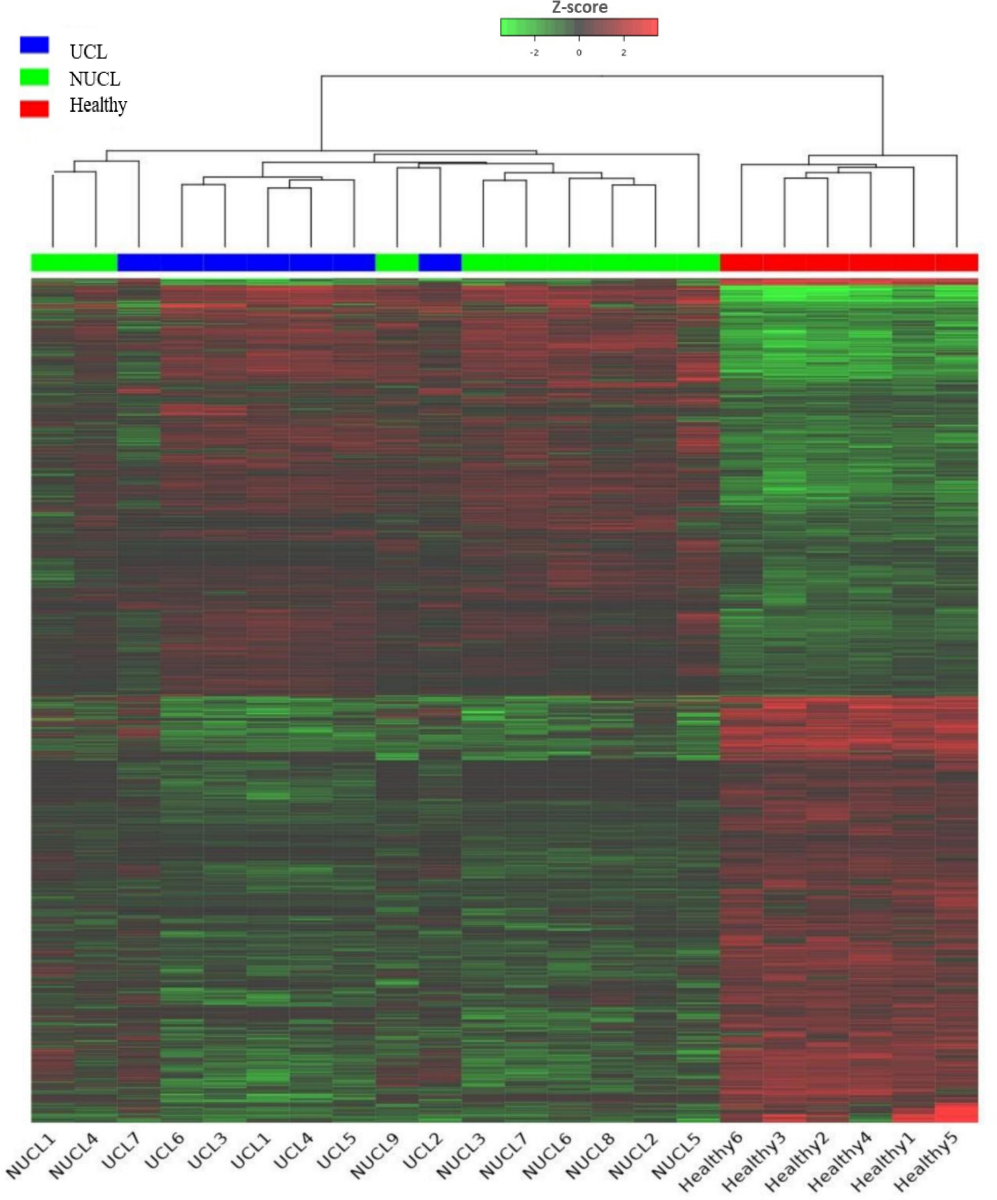
Heatmap showing the expression of all lncRNA genes in CL patients and healthy groups. Each column represents a sample; each row represents an lncRNA. The group belongings are indicated at the top UCL (blue), NUCL (green), and healthy groups (red). Z scores of cpm read counts were used, and the color scale corresponds to the score and represents the level of expression of the lncRNAs hierarchically grouped. Green indicates lower expression, whereas red indicates higher expression.

**Figure 2:**
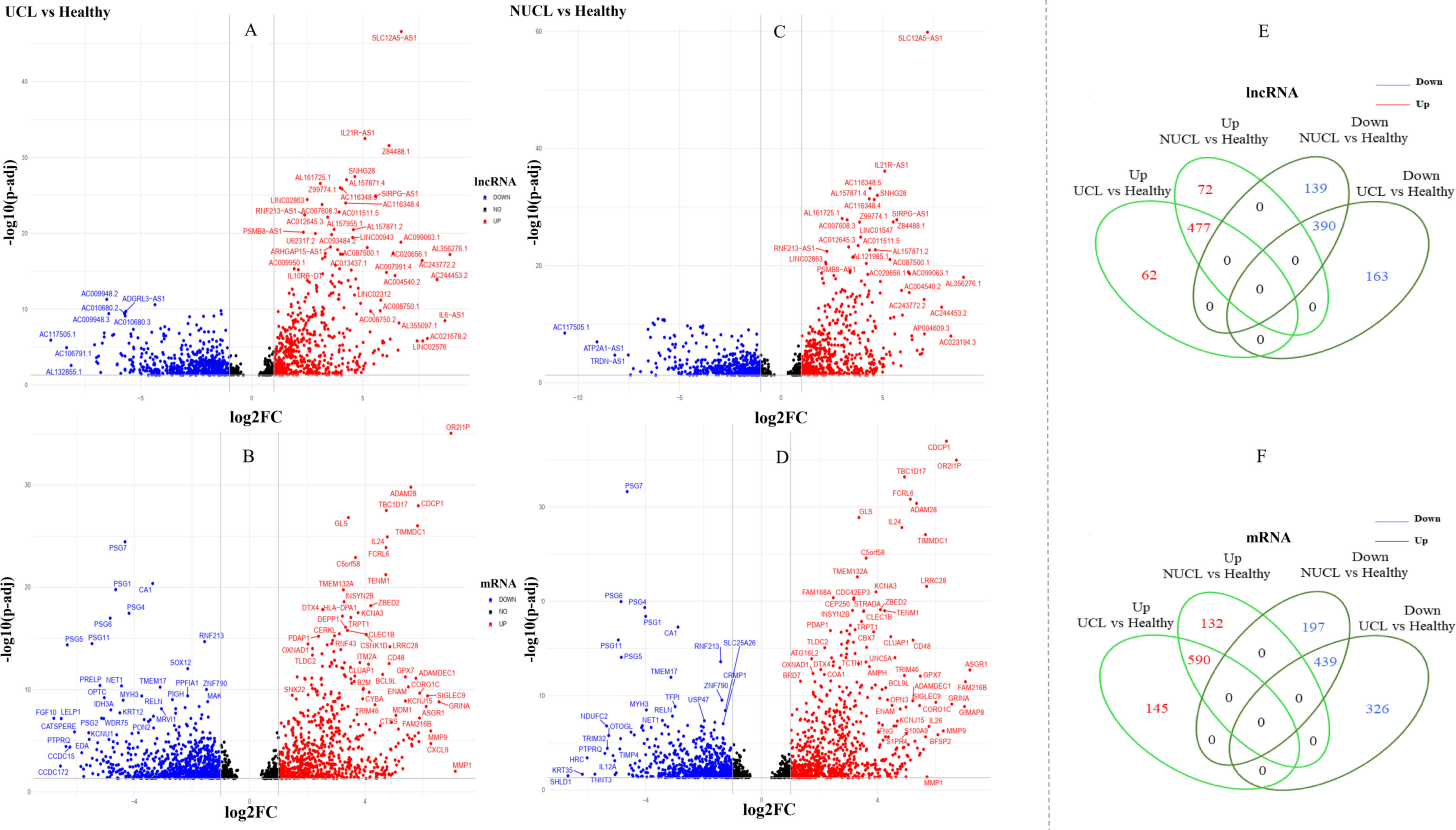
Differential expression gene analysis. **(A–D)** show volcano plots of the expressed mRNAs and lncRNAs in two groups, UCL and NUCL, when compared to a healthy group. The *X*-axis displays Log2FC, while the *Y*-axis shows −log10 of an adjusted *p*-value. **(E, F)** show a Venn diagram of the expressed lncRNAs and mRNAs between the two groups, UCL and NUCL. FC, fold change; UCL, ulcerative CL; NUCL, non-ulcerated CL.

Based on our analysis, a total of 1,533 lncRNAs were identified as significantly expressed genes. The heatmap plot (**Figure 1**) depicts that the expression pattern of annotated lncRNAs is distinct between CL groups (UCL and NUCL) and healthy groups. However, there is no clear distinction between UCL and NUCL groups. In the UCL group compared to the healthy group, 1,260 and 1,765 statistically significant expressed genes (adjusted *p*-value <0.05) were annotated as lncRNA and mRNA genes, respectively (**Supplementary Table S3**). Among the lncRNA gene list, the differential analysis revealed that the expression of 546 genes was increased and that of 554 genes was decreased (**Figure 2A**), whereas in the mRNA group, 736 and 787 genes were determined as up- and downregulated genes, respectively (**Figure 2B**).

In the NUCL group versus the healthy group, a total of 1,240 statistically significant expressed genes (adjusted *p*-value <0.05) were identified as lncRNA genes, in which 549 and 529 genes were up- and downregulated, respectively (**Figure 2C**). Additionally, 1,606 genes were annotated as mRNAs, with 722 upregulated and 636 downregulated genes being identified (**Figure 2D**).

The Venn diagram showed that there are obvious shared genes between the two patient groups (UCL and NUCL) encompassing 967 lncRNAs and 1,192 mRNAs. Among shared lncRNA genes, 477 and 390 genes were up- and downregulated, respectively (**Figure 2E**), while 590 and 439 up- and downregulated genes were identified among shared mRNA genes, respectively (**Figure 2F**). The RNA-seq datasets of each patient group also revealed unique expressed lncRNAs and mRNAs as compared to healthy samples (**Figures 2E, F**; **Supplementary Table S3**).

### 3.2 Co-expression network construction and module identification

**Figure 3:**
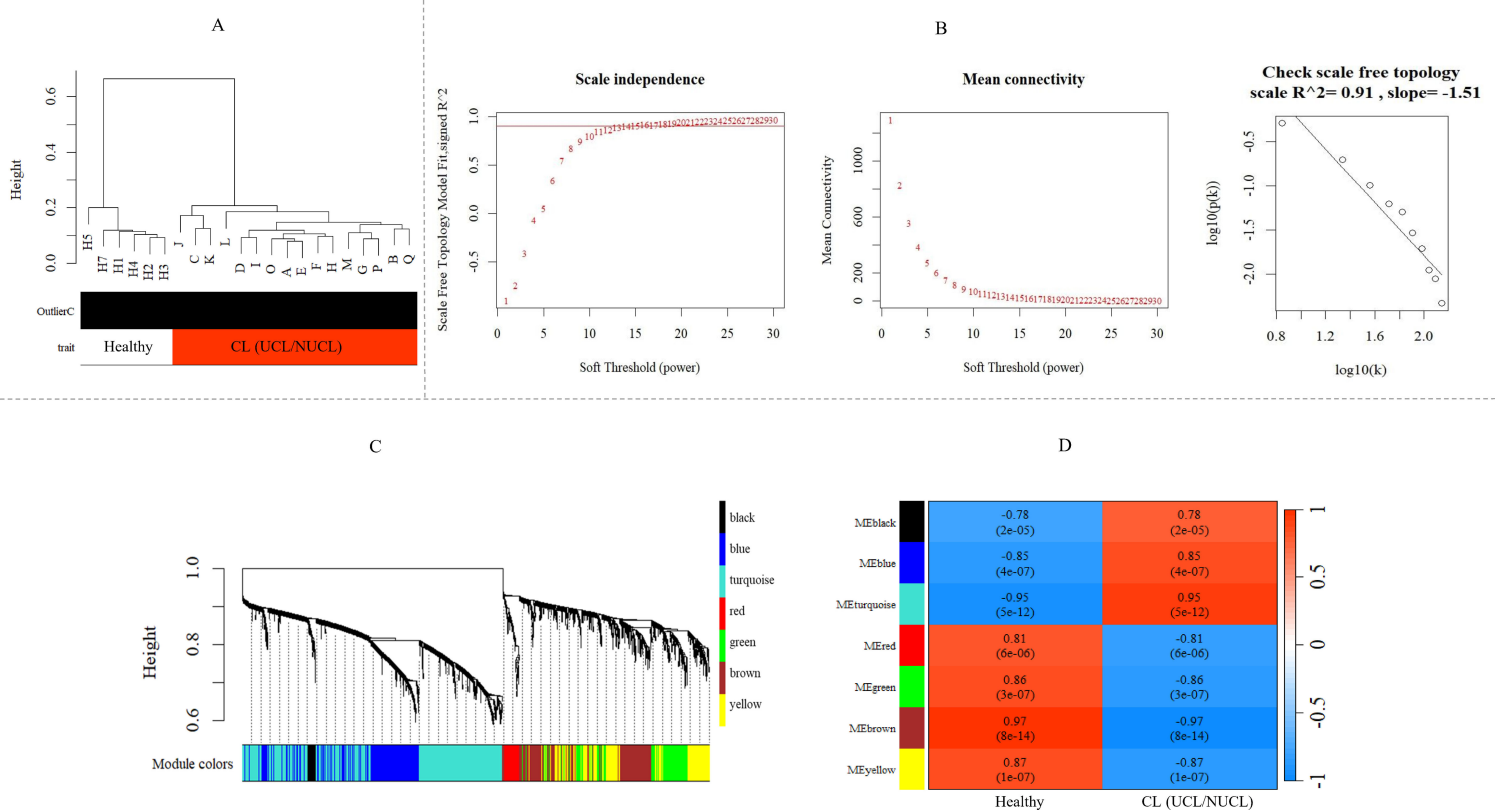
WGCNA of mRNA and lncRNA genes revealed gene co-expression modules in CL patients. **(A)** Sample dendrogram clustering to detect outliers in the GSE216638 dataset. **(B)** Freedom relative weight choice related co-expression modules. The soft thresholding powers are presented by numbers in the plots. The plot of connectivity distribution and the scale-free topology check when β = 15. Plot log–log shows a scale-free topology fit (*R*²) of 0.91. WGCNA, weighted gene co-expression network analysis. **(C)** Hierarchical clustering dendrogram of the shared mRNA and lncRNA genes between UCL and NUCL groups. The branches and color bands represent the assigned modules. **(D)** The module–trait relationship. The color scale corresponds to the scores representing the correlation levels between module eigengenes and the disease. A strong positive and negative correlation indicated by red and blue colors, respectively. The corresponding correlation and *p*-value are shown in each cell. CL, cutaneous leishmaniasis; UCL, ulcerative CL; NUCL, non-ulcerated CL.

Next, we assessed potential co-expression networks and modules formed by shared DEGs (adjusted *p*-value < 0.05) that are common between the two clinical forms of CL, i.e., UCL and NUCL. After identifying the shared DE lncRNAs and mRNAs, WGCNAs on the whole transcriptome data were performed to explore the co-expressed gene networks as well as mRNAs and lncRNAs of interest. The parameter analysis related to WGCNA of consensus genes is presented in **Figures 3A, B**.

As shown in **Figure 3C**, seven modules were assigned based on the hierarchical clustering and the WGCNA cutreeDynamic function in the shared genes between UCL and NUCL groups. A unique color label was assigned to each module. The turquoise module was the largest UCL–NUCL-related module containing 335 lncRNA and 426 mRNA genes, while the smallest module was the black module encompassing 16 lncRNA and 22 mRNA genes. Additionally, as most genes distributed in the turquoise module were upregulated, it was classified as an upregulated module. Details of all identified modules are provided in **Table 1**.

**Table 1:** Details of the assigned modules by WGCNA in the UCL–NUCL group.

Type	Module	Genes	lncRNA genes		mRNA genes		Total DEGs (Log_2_FC) UCL		Total DEGs (Log_2_FC) NUCL	
			*N*	%	*N*	%	Upregulation	Downregulation	Upregulation	Downregulation
**UCL–NUCL shared genes**	Turquoise	761	335	44	426	56	589	114	585	110
	Blue	405	199	49.1	206	50.9	280	86	263	83
	Brown	334	154	46.1	180	53.9	84	216	81	206
	Yellow	321	117	36.4	204	63.6	46	233	44	228
	Green	230	106	46.1	124	53.9	40	160	39	153
	Red	70	40	57.14	30	42.86	21	44	20	44
	Black	38	16	42.11	22	57.89	20	7	21	6

**Figure 4:**
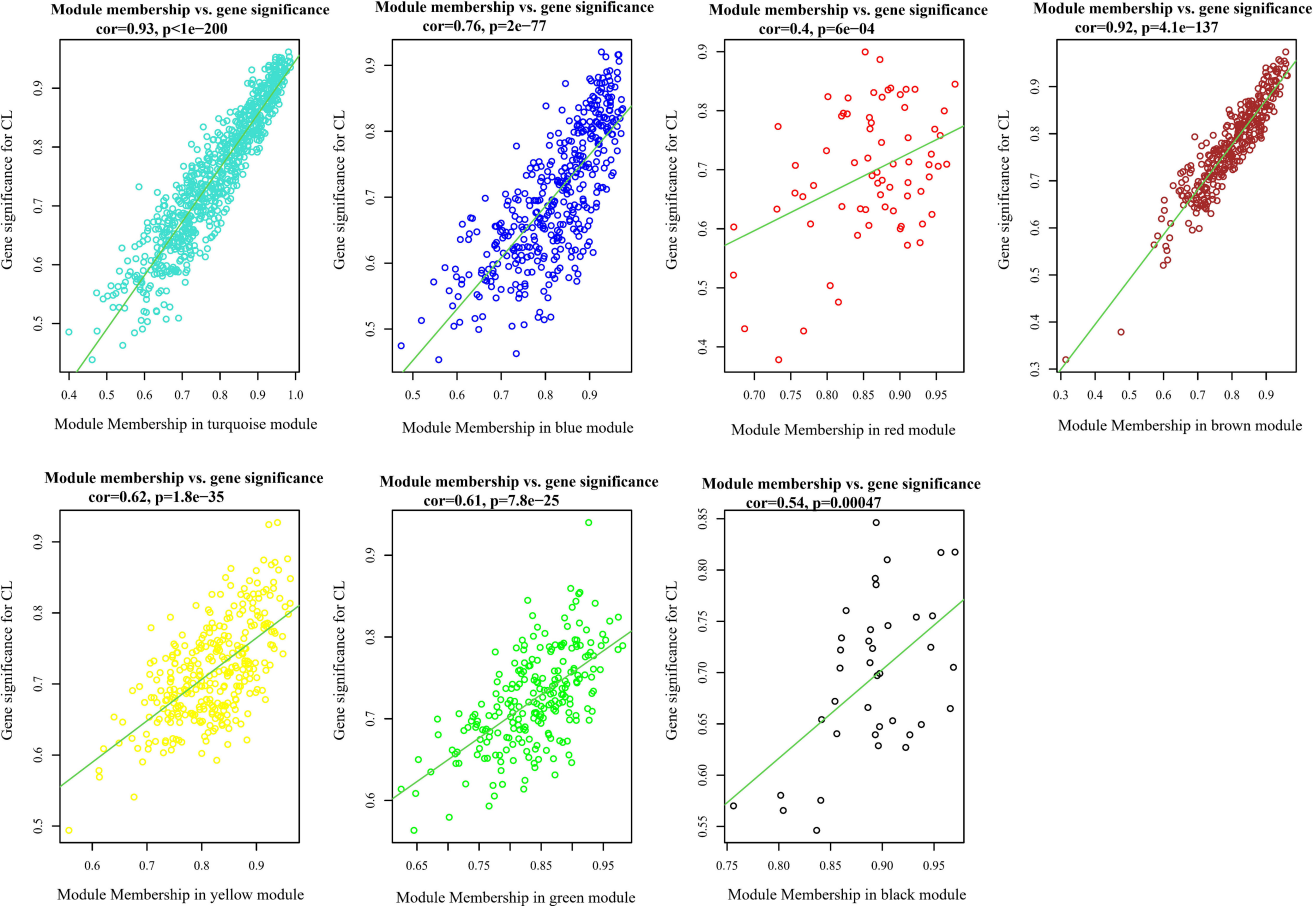
Scatter plots of gene significance (GS) vs. module membership (MM) in all assigned modules through WGCNA. CL, cutaneous leishmaniasis; UCL, ulcerative CL; NUCL, non-ulcerated CL.

**Figure 5:**
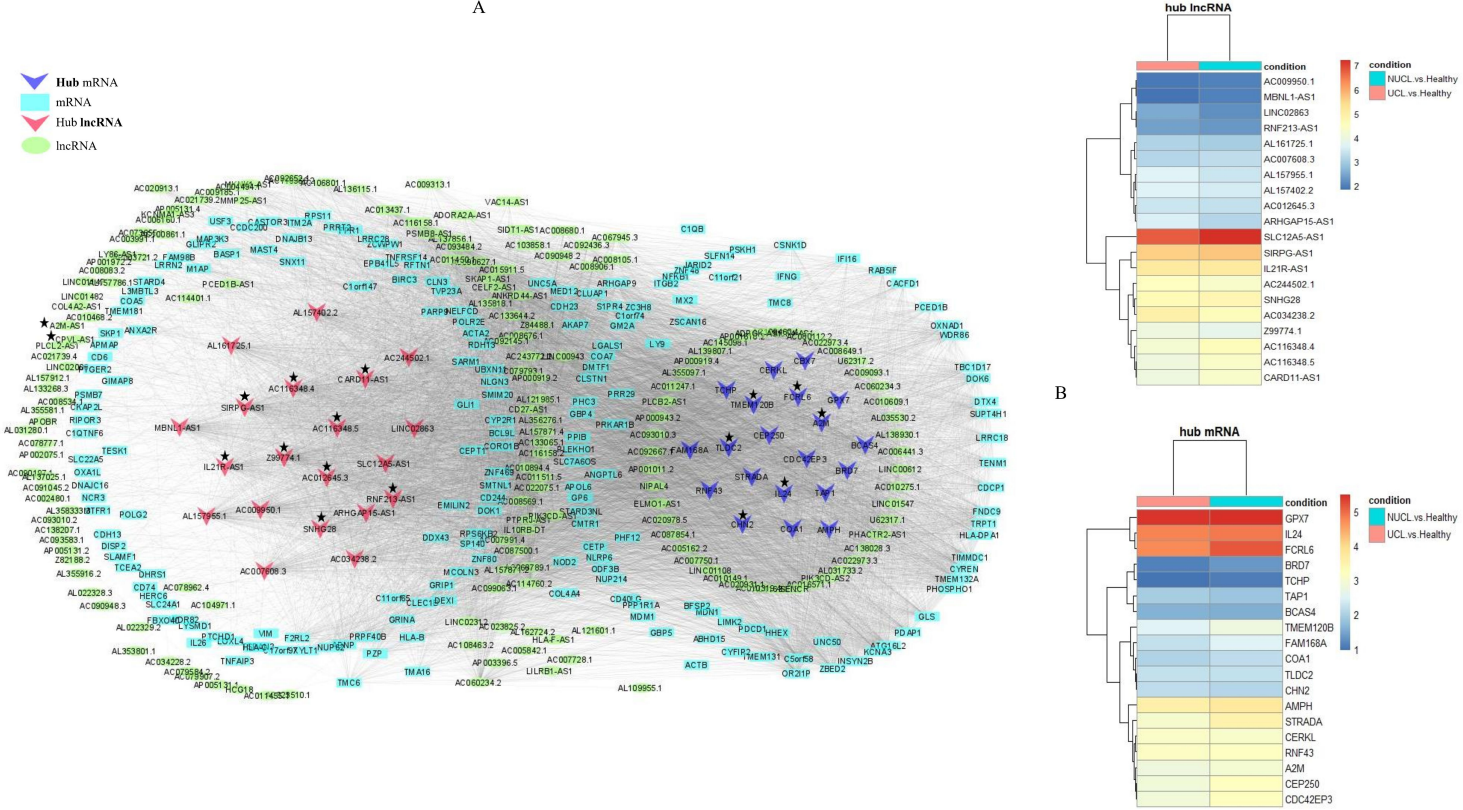
The regulatory network of coding–non-coding genes is distributed in the turquoise module as the key module. **(A)** The lncRNA–mRNA gene network for differentially expressed genes in the turquoise module. The ellipses represent lncRNAs, rectangles represent mRNAs, and inverted triangles represent hub lncRNAs and mRNAs. The turquoise and red colors were used for lncRNAs and mRNAs node coloring, respectively. The hub lncRNA and mRNA pairs (cis or trans) highlighted in the study are presented by asterisks. **(B)** Heatmap of hub DE genes were identified in the turquoise module. The log2FC values of mRNA and lncRNA genes were represented by color codes: red for high values and blue for low values. FC, fold change.

Analysis of the association between modules and the disease provided information on modules that were most affected in the skin lesions of CL patients infected with *L. tropica*. This analysis showed a significantly high positive and negative correlation in most identified modules in the UCL–NUCL group. Among all modules, the highest significant positive correlation was related to the turquoise module, while brown was determined as a module with the highest negative correlation. Graphical visualization of the correlation values and their corresponding *p*-values is illustrated in **Figure 3D**. In subsequent analyses, based on GS-MM correlation results, the turquoise module with the highest correlation value (*r* = 0.93 and *p*-value <0.05) was determined as the key module in the UCL–NUCL group (**Figure 4**). The gene network visualization of co-expressed genes in the key module is presented in **Figure 5A**. Then, we focused only on the key module to identify hub genes and co-expressed pairs of mRNAs and lncRNAs. The top 20 mRNA and lncRNA genes met the eligibility criteria of a minimum absolute threshold value of 0.6 for MM and GS, and the highest value for degree was selected as UCL–NUCL associated hub genes in the key module (**Figure 5B**). Out of the 20 selected hub lncRNAs, 11 were identified as novel lncRNA genes (**Table 2**). Additionally, based on the subcellular location prediction, the hub lncRNAs mainly reached the highest score for nuclear and cytoplasmic localization. However, the localization of some lncRNAs remains unknown (**Table 2**).

**Table 2:** The potential trans-target of hub lncRNAs identified among correlated lncRNA–mRNA pairs in the turquoise module in *L. tropica*-infected patients.

Turquoise module Potential trans-acting lncRNAs									
lncRNAs location ^c1^	lncRNA hub genes	Top co-expressed mRNA genes	TF (*N*)	Co-expressed mRNA genes	lncRNAs location ^c1^	lncRNA hub genes	Top co-expressed mRNA genes	TF (*N*)	Co-expressed mRNA genes
**-**	IL21R-AS1	IL24 FAM168A FCRL6	–	–	Cytoplasm	AC012645.3	IL24 CDC42EP3 CERKL	1	MED12
**-**	SIRPG-AS1	CDC42EP3 IL24 TAP1 FCRL6 TLDC2	–	–	Cytoplasm	AC116348.4	CEP250 TLDC2 IL24 TMEM120B	–	–
Nucleus	CARD11-AS1	TMEM120B	–	–	–	AC116348.5	CEP250 IL24 TLDC2 TMEM120B	–	–
Nucleus	SLC12A5-AS1	FCRL6 FAM168A TBC1D17	1	MED12	Nucleus	Z99774.1	IL24 TAP1 TLDC2	2	MED12 NFKB1
Nucleus	SNHG28	FCRL6 TLDC2 FAM168A BRD7	–	–	Nucleus	AC009950.1	CEP250	–	–
Nucleus	ARHGAP15-AS1	TAP1	–	–	Cytoplasm	AC007608.3	CEP250	–	–
Nucleus	MBNL1-AS1	CDC42EP3	2	MED12 NFKB1	Cytoplasm	AL161725.1	TAP1	1	MED12
Nucleus	RNF213-AS1	TLDC2 TBC1D17	–	–	–	AC244502.1	CDC42EP3 TAP1	–	–
Exosome	LINC02863	–	–	–	–	AL157955.1	TAP1 LRRC18	–	–
**-**	AC034238.2	CEP250	–	–	Cytoplasm	AL157402.2	TAP1 LRRC18	–	–

c1 only prediction scores greater than 0.6 were included.

“-”: not identified (subcellular localization, TF, and co-expressed mRNA genes).

### 3.3 Cis- and trans-interactions

In order to identify the possible regulatory relationships between lncRNAs (cis and/or trans) and their mRNA target genes, we investigated potential cis/trans-acting lncRNAs in the key module. Our search for cis-targets of lncRNAs in the turquoise module led to the discovery of 29 pairs of DE lncRNAs and mRNAs (**Table 3**). Expression of all identified pairs of lncRNA and mRNA genes was increased. It is worth noting that out of the 29 lncRNAs, 21 were novel transcripts and had no annotation in non-coding RNA databases. Nevertheless, we were able to predict their cis-target genes and subcellular localization; we primarily focused on the hub lncRNA–mRNA pairs. The potential trans-target of hub lncRNAs was determined by ranking correlation value, and the top highly correlated pairs (| ***r*** | > 0.95) are presented in **Table 2**. Additionally, the interaction of selected hub lncRNAs with TFs, as the other possible interactor, retrieved from the RNAInter database was identified. As shown in **Table 2**, in trans-interactions of hub lncRNAs–mRNAs, several hub lncRNAs were found to have regulation relationships with the TFs.

**Table 3:** Potential cis-targets of DE lncRNAs related to the turquoise module in the lesion of *L. tropica*-infected patients.

Turquoise module Potential cis-acting lncRNAs					
lncRNAs location ^c1^	lncRNAs	mRNA pair in chromosome vicinity	lncRNAs location ^c1^	lncRNAs	mRNA pair in chromosome vicinity
Nucleus	PIK3CD-AS2	CLSTN1	Nucleus	A2M-AS1	A2M, PZP
Cytoplasm	AC007608.3	NOD2	Cytoplasm	AC116158.1	FAM98B
Cytoplasm	SKAP1-AS1	SNX11	Nucleus	AC106801.1	NIPAL4
Nucleus	LINC01547	ITGB2	Cytoplasm	AC093788.1	TMA16
-	AP004609.3	BCL9L	Cytoplasm	AC092436.3	SMIM20
Nucleus	AP000919.2	EMILIN2	–	AC078889.1	GRIP1
Nucleus	AP000763.4	FAM168A	Cytoplasm	AC068789.1	PPP1R1A
Nucleus	AL137856.1	LY9	Nucleus	AC020931.1	ANGPTL6
Cytoplasm	AL133230.2	RIPOR3	Nucleus	AC007613.1	SNN
Cytoplasm	ADPGK-AS1	BBS4	–	AC015917.2	SARM1
Nucleus	U62317.1	ODF3B	Cytoplasm	Z82188.2	CYTH4
Nucleus	AC008906.1	CAST	Cytoplasm	AGAP2-AS1	MARCHF9
-	AC080112.2	IGFBP4	Cytoplasm	HLA-DQB1-AS1	HLA-DQA1
-	AC005041.3	DOK1	Nucleus	CPVL-AS1	CHN2
Nucleus	AL365361.1	KCNA2			

c1 only prediction scores greater than 0.6 were included.

All mRNA genes distributed in the turquoise module can be considered trans-target genes of the lncRNAs in this module. In line with this, coding genes not found in searching for the cis-target are considered potential trans-targets of correlated DE lncRNAs in the turquoise module. Of the co-regulated pairs of DE lncRNAs and mRNAs in the turquoise module, we identified 12,562 positive and 2,891 negative pairs with strong correlation (|*r*| > 0.8 and *p* < 0.05). Accordingly, most DE lncRNA networks play a role in transcription as trans-acting lncRNAs. Therefore, we primarily focused on the hub lncRNA–mRNA pairs. The potential trans-target of hub lncRNAs was determined by ranking correlation value, and the top highly correlated pairs (|*r*| > 0.95) are presented in **Table 2**. Additionally, the interaction of selected hub lncRNAs with TFs, as the other possible interactor, retrieved from the RNAInter database was identified. As shown in **Table 2**, in trans-interactions of hub lncRNAs–mRNAs, several hub lncRNAs were found to have potential regulation relationships with the TFs. SLC12A5-AS1, MBNL1-AS1, AC012645.3, Z99774.1, and AL161725.1 showed regulation relationships with MED12. Notably, Z99774.1 and MBNL1-AS1 were also found to putatively interact with NFKB1.

### 3.4 Gene ontology and pathway enrichment analysis

**Figure 6:**
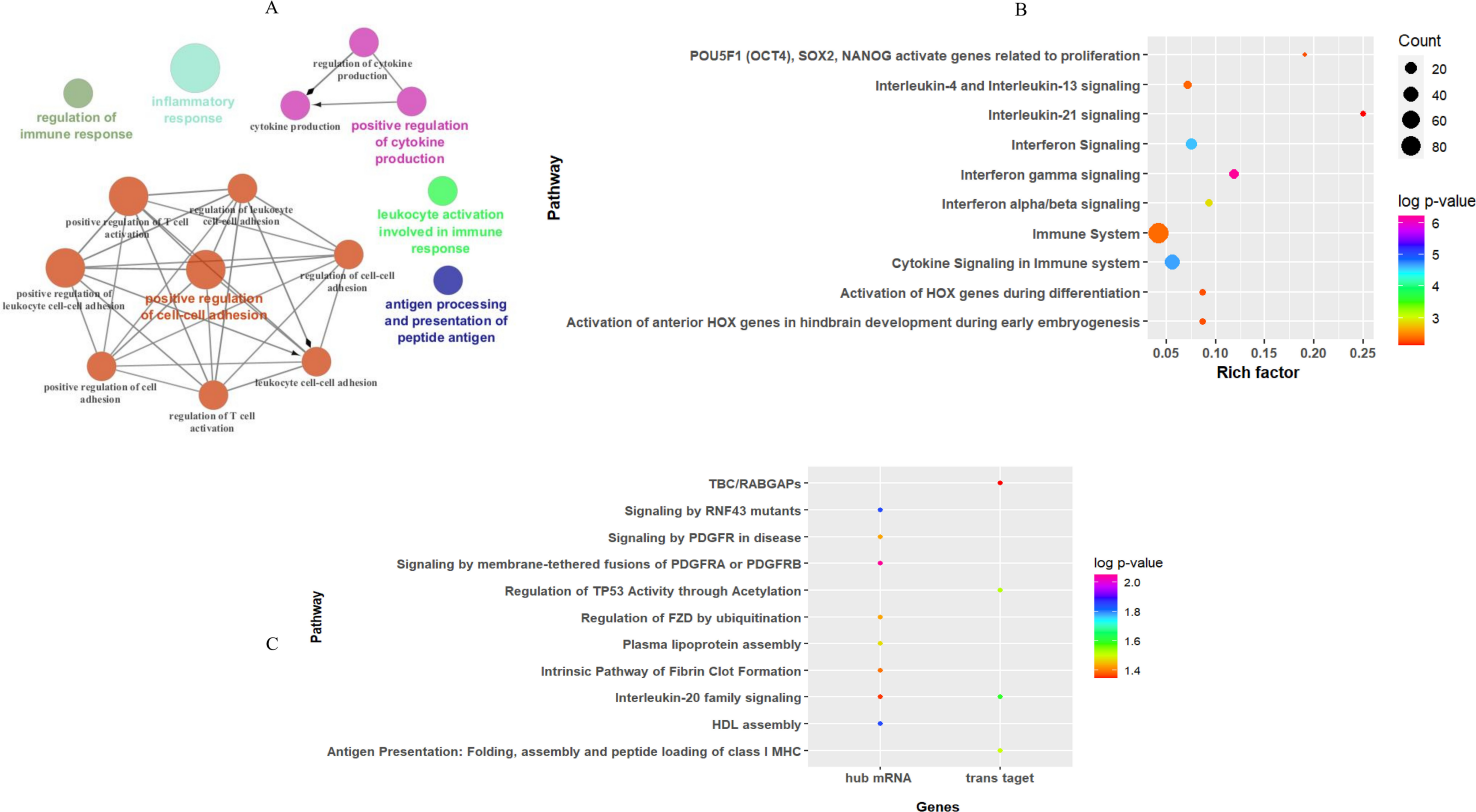
Functional enrichment analysis results for mRNAs based on WGCN. **(A)** The entries of GO enrichment analysis in the turquoise module. Top 10 entries of the Reactome pathway enriched by **(B)** all mRNA genes, and **(C)** hub mRNA along with trans-target genes of hub lncRNAs in the turquoise module.

Enrichment analysis of the turquoise module revealed that 87 GO biological processes (six clusters) and 36 Reactome pathways were significantly enriched. GO annotation of mRNAs in key modules indicated that inflammatory response (GO: 0006954), positive regulation of cell–cell adhesion (GO: 0022409), and antigen processing and presentation of peptide antigen (GO: 0048002) were among the top terms of enrichment in the biological process category (**Figure 6A**). The Reactome pathways were mainly associated with “interferon gamma signaling”, “cytokine signaling in the immune system”, and “interferon signaling” in the key module (**Figure 6B**). These enrichment results were consistent with the pathogenesis of CL. The details of entries for GO annotation and pathway enrichment are reported in **Supplementary Table S4**.

In addition, Reactome pathway analysis of hub mRNA genes in the key module indicated that “signaling by membrane-tethered fusions of PDGFRA or PDGFRB”, “HDL assembly”, and “regulation of FZD by ubiquitination” were the top enriched pathways (**Figure 6C**).

Prediction of pathways enriched by hub lncRNA trans-target genes demonstrated that they were enriched in three pathways: “interleukin-20 family signaling”, “regulation of TP53 activity through acetylation”, and “antigen presentation: folding, assembly, and peptide loading of class I MHC” (**Figure 6C**), while cis-target genes of DE lncRNA in the key module were mainly involved in four pathways: “extracellular matrix organization”, “Toll-Like Receptor 4 (TLR4) cascade”, “translocation of ZAP-70 to immunological synapse”, and “interferon-gamma signaling”.

### 3.5 Validation of DE lncRNA and mRNA genes related to the key module

**Figure 7:**
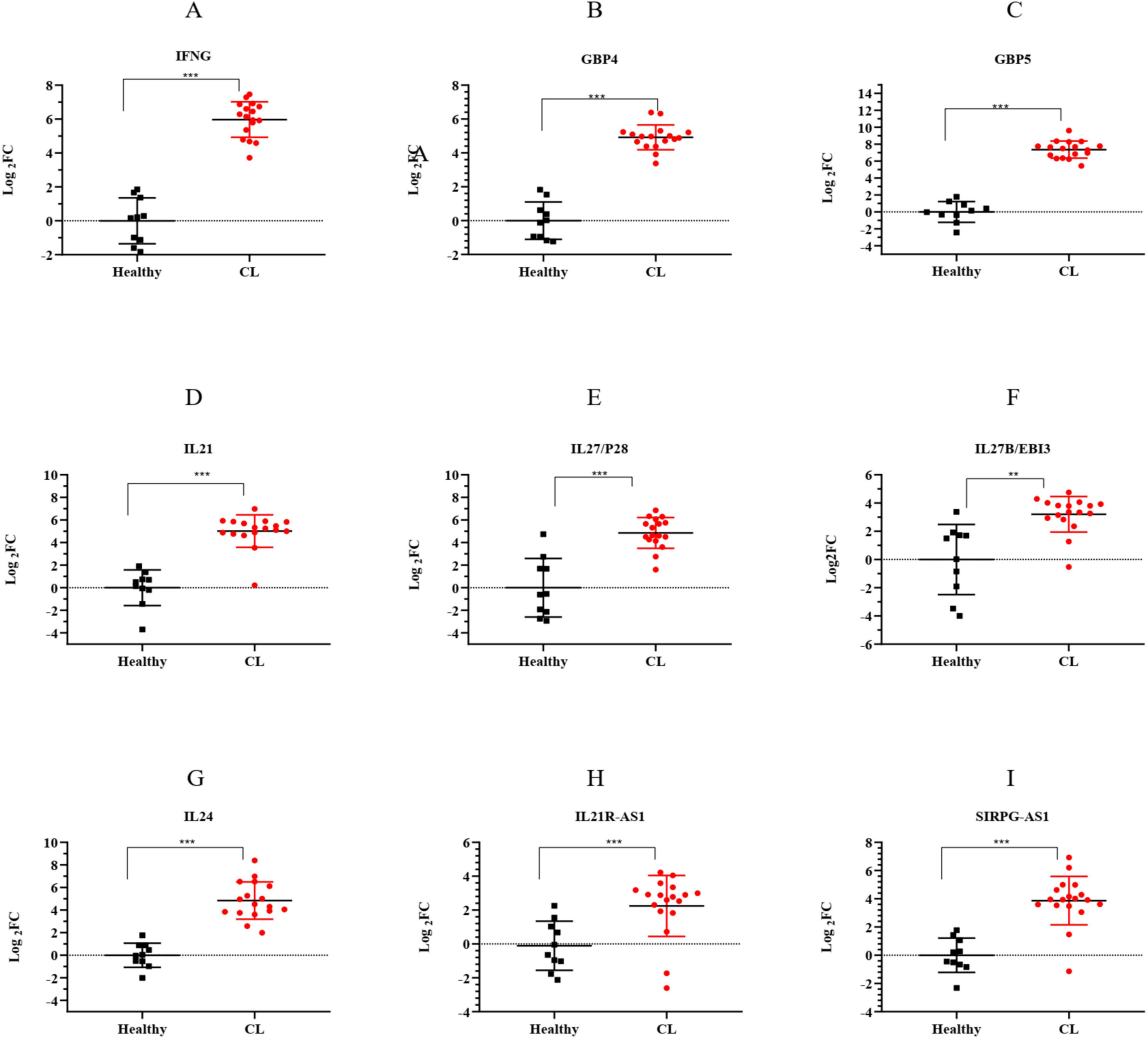
The expression levels of selected lncRNA and mRNA genes from the turquoise module between *L. tropica*-infected patients and the healthy group as determined by qRT-PCR. **(A)** IFNG; **(B)** GBP4; **(C)** GBP5; **(D)** IL21; **(E)** IL27/P28; **(F)** IL27B/EBI3; **(G)** IL24; **(H)** IL21R-AS1; and **(I)** SIRPG-AS1; *p*-values obtained using the Mann–Whitney *U* test are shown (**p < 0.01, and ***p < 0.001). GAPDH was used as a reference gene. The results were shown as the mean ± SD of duplicate measurements. FC, fold change.

To validate the accuracy of RNA-seq data, the expression of seven mRNA genes was assessed in 18 CL patients and 10 healthy volunteers using the qRT-PCR method. The selected genes were related to the top two entries of the Reactome pathway enriched in the key module, interferon gamma signaling and cytokine signaling, with high expression. The genes were involved in interferon gamma signaling including Interferon-gamma (IFNG), Guanylate-binding protein (GBP)4, and GBP5. Interleukin21 (IL21), IL27 (P28/EBI3), and IL24 were related to cytokine signaling. Furthermore, the expression of two lncRNAs, IL21R-AS1 and SIRPG-AS1, which were among the highly expressed hub lncRNAs targeting IL24, as a hub mRNA, was evaluated. As shown in **Figure 7**, we compared the expression of these genes in patients infected with *L. tropica* to those of a healthy group. The results showed that all genes had increased expression (log2FC > 1, *p* < 0.05) in the infected patients compared to the healthy group. Overall, the relative expression analysis validated the significant upregulation of selected genes in CL infection caused by *L. tropica* that supported the reproducibility of the included RNA-seq data. These findings provide further evidence of the involvement of “interferon-gamma signaling” and “cytokine signaling” in CL infection.

## 4 Discussion

Studying host molecular signatures in the context of host–*Leishmania* interactions has gained momentum with the advancement of omics analyses. These studies have highlighted a potential role for host transcriptional dysregulation in leishmaniasis infection with protein-coding genes (Maretti-Mira et al., 2012; Gardinassi et al., 2016; Fakiola et al., 2019; Adriaensen et al., 2020; Masoudzadeh et al., 2020b; Farias Amorim et al., 2021). Besides these studies, a few recent reports have explored the role of other biological factors like lncRNAs during leishmaniasis (Fernandes et al., 2022; Maruyama et al., 2022; Almeida et al., 2023), which contributed to our understanding of the regulatory network involved in leishmaniasis. Therefore, exploring coding–non-coding signatures and translational implications of the diverse regulation could be a promising focus for future research, as it may help address the challenges posed by this complex disease.

To our knowledge, this study is the first to investigate the profile of lncRNAs and their potential connections with mRNAs in the lesion of CL patients infected with *L. tropica* using a systems biology approach. In our analyses, WGCNA enabled us to gain an overview of the system-level functionality of the significantly expressed gene in the whole transcriptome dataset and find key biological processes affected by the interplay between lncRNAs and mRNAs. This study leverages the strength of WGCNA, which prioritizes connections over discrete components and avoids misinterpretation about the role of specific pathways in a particular condition, to provide more reliable and comprehensive results than those obtained based solely on mean expression (Weighill et al., 2021; Panditrao et al., 2022).

According to WGCNA of shared lncRNA and mRNA genes in our dataset, seven modules associated with CL due to *L. tropica* were identified. Of note, a module with a strong positive correlation value, namely, turquoise, was highlighted as the key module. Through functional enrichment analysis of the key module, we found that the enriched terms, as expected, were mainly associated with immune pathways like “interferon-gamma signaling” and “cytokine signaling”. Both of the enriched pathways have been recognized as key mechanisms involved in determining the outcome of leishmaniasis infection (Scott and Novais, 2016).

Hence, both coding and non-coding genes were classified in the turquoise module; we analyzed the co-expression of lncRNA and mRNA genes to identify hub lncRNA and mRNA genes and infer the possible function of lncRNAs. The hub genes might represent the main lncRNA and mRNA genes that influence the functionality of the key module. Additionally, we explored the type of regulatory interactions (cis or trans) of the identified hub lncRNAs.

Among the identified mRNA hubs, IL24, TBC/LysM-associated domain containing 2 (C20ORF118 or TLDC2), and Transmembrane Protein 120B (TMEM120B) genes were the top three genes that had the highest connection with other genes. These genes are found as the potential trans-target of 9 out of 20 hub lncRNAs in our study (**Table 2**). IL-24 belongs to the IL-20 cytokine subfamily and is predominantly expressed in T cells and macrophages (Dabitao et al., 2018). The pro-inflammatory cytokine acts as a regulator of keratinocytes in the skin and mediates tissue repair in injured barrier epithelial cells (Sa et al., 2007; Liu et al., 2023). However, biologic actions and expression patterns of IL-24 are rather understudied in leishmaniasis, and based on the role of IL-24 in wound healing, it can be reasonable to consider a protective effect for IL-24 in CL.

On the IL-20 cytokine subfamily, we found a co-expressed pair of IL21R-AS1/IL24, in which expression of both genes was increased based on RNA-seq and qRT-PCR data. Increased levels of IL21R-AS1 gene in our data might act as an activator of IL24-dependent signaling. Different from our results, a recent study demonstrated the downregulation of IL21R-AS1 and the corresponding cis-target, namely, IL21R in *L. infantum*-infected THP1 (Almeida et al., 2023). In other studies, IL21R-AS1 was reported as a candidate coronary artery disease biomarker (Cai et al., 2016) and as an immune-related lncRNA predicting cervical squamous cell carcinoma prognosis (Lv et al., 2022).

The SIRPG-AS1/IL24 was another identified co-expressed pair on the IL-20 cytokine subfamily in our study, which were upregulated based on RNA-seq and qRT-PCR results. The SIRPG-AS1 is poorly studied, with one report related to potential prognostic lncRNAs in the squamous cell carcinoma type (Tian et al., 2020). Furthermore, in our data, IL24 was also found to be the predicted trans-regulating target of four novel hub lncRNAs, namely, AC012645.3, AC116348.4, AC116348.5, and Z99774.1. No study has yet investigated the potential function of these lncRNAs. Interestingly, two of those upregulated lncRNAs, namely, AC012645.3 and Z99774.1, were also predicted to target TFs. The upregulated TFs were Mediator Complex Subunit 12 (MED12) and nuclear factor kappa B subunit 1 (NFKB1). MED12 is involved in regulating essential steps of transcription, and its deregulation was linked to human cancers (Zhang et al., 2020), while NFKB1 plays a role in macrophage polarization and innate immune memory responses (Porta et al., 2009). In the cases of leishmaniasis, interrupted TF-mediated regulation has been described (Lecoeur et al., 2022). For instance, *L. amazonensis* interferes with NF-κB TF function through downregulation of RELA, NFKB1, and NFKB2 during early infection in BMDM (Lecoeur et al., 2020). Although a recent study predicted several lncRNA–TF interactions in *L. infantum* infection (Maruyama et al., 2022), the role of lncRNAs in regulating TFs and the underlying molecular mechanisms remains to be elucidated in leishmaniasis.

In addition to IL24, TLDC2 and TMEM120B were also among the top three hub mRNA genes in our analysis. TLDC2 is known as a member of the TLDCs family, which has an important role in the oxidative stress response (Finelli and Oliver, 2017). TMEM120B is expressed in fat and has a regulatory role in the differentiation of adipocytes (Batrakou et al., 2015). However, the potential role of these genes in infectious diseases, including leishmaniasis, remains unknown. Here, TLDC2 and TMEM120B mRNA hub genes were found to be co-expressed with several hub lncRNAs, including SIRPG-AS1, CARD11-AS1, SNHG28, RNF213-AS1, AC116348.5, and Z99774.1. While the role of these upregulated lncRNAs is unclear in leishmaniasis, SNHG28 is recognized as a type of lncRNA, named small nucleolar RNA host gene (SNHG), which is involved in the development and aggressiveness of cancer. The SNHG family has been suggested as a potential prognostic and immunotherapeutic response biomarker (Shi et al., 2020; Zimta et al., 2020; Zheng et al., 2023). In the context of leishmaniasis, SNHG29, a member of the SNHG family, was determined as hub lncRNA targeting S100A8 mRNA in *L. braziliensis* skin infection. This study suggested that the upregulated pair may increase the recruitment of neutrophils to the site of the infection (Almeida et al., 2023). Here, we also found another predicted target for SNHG28 termed Fc receptor-like 6 (FCRL6). FCRL6 is an immunoregulator gene in cytotoxic T and NK cells, and the elevated level of this gene and its potential mechanistic role represented this gene as a favorable biomarker and therapeutic target in cancer (Davis, 2020). Expression of FCRL6 as a hub mRNA gene was also upregulated in our study. Based on the role of FCRL6 in cytotoxicity, this result may imply a potential pathogenic role for the predicted co-expressed pair in the CL infection.

Our study has also identified potential cis-acting lncRNAs, most of which were novel transcripts with unknown functions (**Table 3**). A noteworthy instance of annotated cis-acting lncRNAs, A2M-AS1, targeted alpha 2-Macroglobulin (A2M), a hub mRNA. A2M is known as a human protease inhibitor, which is potentially involved in host defense and infection (Vandooren and Itoh, 2021). Evidence from recent studies described A2M-AS1 as a prognostic biomarker in different cancers (Zhou et al., 2019; Fang et al., 2020; Ye and Jin, 2021; Qiu et al., 2022). However, the significance of this upregulated pair in CL has not been studied previously; we speculated that this overexpressed pair would be associated with CL progression by boosting pro-inflammatory cytokine levels. The other predicted cis-acting lncRNA, CPVL-AS1, was linked to the hub mRNA gene CHN2 chimerin 2 (CHN2). CHN2 negatively regulates Rac GTPase activity, which has a role in the control of actin cytoskeleton dynamics. These genes play a role in cell proliferation, adhesion, and T-cell activation. CHN2 deregulation has been linked to mental disorders and cancers (Wertheimer et al., 2012; Barrio-Real et al., 2013; Finalet Ferreiro et al., 2014). However, the function of CPVL-AS1 lncRNAs is currently unknown. Investigating the functional roles of the predicted cis-acting lncRNAs and their corresponding mRNA gene pairs can open up new avenues for studying regulatory networks and the intricate biological mechanisms related to CL.

In addition, the current study validated the expression of some mRNA genes associated with top enriched pathways, namely, “interferon gamma signaling” and “cytokine signaling”, using the qRT-PCR method. The qRT-PCR results showed overexpression of IFNG, GBP genes (GPB-4 and GBP-5), IL21, and IL27 (P28/EBI3) in CL patients infected with *L. tropica.* IFN-γ, as a critical cytokine, exerts its function in immunoprotection against *Leishmania* through modulating the activity of macrophage and Th1 response (Kima and Soong, 2013). Elevated levels of this cytokine have been documented following CL infection (Galgamuwa et al., 2019; Farias Amorim et al., 2021). Furthermore, GBP genes are known as a family of interferon-inducible dynamin-like GTPases and regulators of immunity to infection. They are among the most frequent IFN-stimulated genes (ISGs) (Kresse et al., 2008; Schoggins, 2019; Tretina et al., 2019). Upregulation of GBP (GBP1–6) genes was seen in CL patients infected with *L. braziliensis* (Farias Amorim et al., 2021). Similarly, we observed increased levels of IFNG, GBP4, and GBP5, as measured by RNA-seq and qRT-PCR, within *L. tropica* CL lesions. However, the role of GBP genes during CL in human is not well understood, and increased levels of IFN-γ and GBP genes could possibly reflect their favorable contribution in eliciting immunity against CL.

Regarding the expression of IL21 and IL27, there is ample evidence showing that IL-21 is involved in inflammation and tissue damage by increasing the effector phase of T-cell responses (Monteleone et al., 2008). However, there is a dearth of studies evaluating the involvement of this cytokine in leishmaniasis. These studies reported the expression of IL21 in the immune response against CL and VL diseases (Espitia et al., 2010; Bollig et al., 2012; Costa et al., 2015; Kong et al., 2017; Khatonier et al., 2018). In addition, Costa et al. showed that IL21 expression has a positive correlation with IFN-γ and IL27 expression in CL caused by *L. braziliensis* (Costa et al., 2015). Here, we found an upregulation of IL21 expression within active skin lesions. The combined evidence of the studies tends to suggest that IL-21 possibly promotes the disease progression. Another studied cytokine, IL-27, has a function in regulating Th1, Th2, and Th17 immune responses (Villarino et al., 2003; Batten et al., 2006). In the context of leishmaniasis, IL-27 exerts its function on T cells in a species-specific manner. This cytokine was found to have a protective role in the early stage of *L. major* infection (Anderson et al., 2009). In contrast, the immunosuppressive effect of IL-27 in response to some species like *L. infantum* and *L. donovani* has been documented (Rosas et al., 2006; Barreto-de-Souza et al., 2015; Quirino et al., 2016; Jafarzadeh et al., 2020). The upregulated IL27 gene in our study may suggest a pathogenic role for IL27 like IL21 in the *L. tropica* CL lesion. Taken together, this evidence indicates that the genes in the key module determined by WGCNA might represent a potential target that influences the clinical outcome of patients with CL due to *L. tropica*.

## 5 Conclusion

In conclusion, the present study demonstrated the deregulation of lncRNAs in the transcriptional perturbations induced by *L. tropica* infection in patients with CL. Analyzing the integrated expression profiles of lncRNAs and mRNAs suggested a potential function for lncRNAs in CL patients. Moreover, we proposed a set of potential hub coding–noncoding genes that may be valuable for future studies aimed at the development of effective diagnostic or therapeutic strategies in CL.

## Data Availability

The original contributions presented in the study are included in the article/**Supplementary Material**. Further inquiries can be directed to the corresponding authors.

## Supplementary material

Supplementary Table 4 Significant gene ontology-biological processes and Reactome pathways in key module (turquoise).

## References

[B1] Adriaensen W. Cuypers B. Cordero C. F. Mengasha B. Blesson S. Cnops L.. (2020). Host transcriptomic signature as alternative test-of-cure in visceral leishmaniasis patients co-infected with HIV. EBioMedicine. 55, 102748. doi: 10.1016/j.ebiom.2020.102748 32361248 PMC7195535

[B2] Almeida M. C. Felix J. S. Lopes M. de Athayde F. R. F. Troiano J. A. Scaramele N. F.. (2023). Co-expression analysis of lncRNA and mRNA suggests a role for ncRNA-mediated regulation of host-parasite interactions in primary skin lesions of patients with American tegumentary leishmaniasis. Acta Trop. 245, 106966. doi: 10.1016/j.actatropica.2023.106966 37302689

[B3] Amorim C. F. Novais F. O. Nguyen B. T. Misic A. M. Carvalho L. P. Carvalho E. M.. (2019). Variable gene expression and parasite load predict treatment outcome in cutaneous leishmaniasis. Sci. Transl. Med. 11, eaax4204. doi: 10.1126/scitranslmed.aax4204 31748229 PMC7068779

[B4] Anders S. Pyl P. T. Huber W. (2015). HTSeq—a Python framework to work with high-throughput sequencing data. bioinformatics. 31, 166–169. doi: 10.1093/bioinformatics/btu638 25260700 PMC4287950

[B5] Anderson C. F. Stumhofer J. S. Hunter C. A. Sacks D. (2009). IL-27 regulates IL-10 and IL-17 from CD4+ cells in nonhealing Leishmania major infection. J. Immunol. 183, 4619–4627. doi: 10.4049/jimmunol.0804024 19748991 PMC2749572

[B6] Bañuls A. L. Bastien P. Pomares C. Arevalo J. Fisa R. Hide M. (2011). Clinical pleiomorphism in human leishmaniases, with special mention of asymptomatic infection. Clin. Microbiol. Infect. 17, 1451–1461. doi: 10.1111/j.1469-0691.2011.03640.x 21933304

[B7] Barreto-de-Souza V. Ferreira P. L. de Carvalho Vivarini A. Calegari-Silva T. Soares D. C. Regis E. G.. (2015). IL-27 enhances Leishmania amazonensis infection via ds-RNA dependent kinase (PKR) and IL-10 signaling. Immunobiology. 220, 437–444. doi: 10.1016/j.imbio.2014.11.006 25466588

[B8] Barrio-Real L. Barrueco M. González-Sarmiento R. Caloca M. J. (2013). Association of a novel polymorphism of the β2-chimaerin gene (CHN2) with smoking. J. Invest. Med. 61, 1129–1131. doi: 10.2310/JIM.0b013e3182a32ff9 23941981

[B9] Batrakou D. G. de Las Heras J. I. Czapiewski R. Mouras R. Schirmer E. C. (2015). TMEM120A and B: nuclear envelope transmembrane proteins important for adipocyte differentiation. PloS One 10, e0127712. doi: 10.1371/journal.pone.0127712 26024229 PMC4449205

[B10] Batten M. Li J. Yi S. Kljavin N. M. Danilenko D. M. Lucas S.. (2006). Interleukin 27 limits autoimmune encephalomyelitis by suppressing the development of interleukin 17-producing T cells. Nat. Immunol. 7, 929–936. doi: 10.1038/ni1375 16906167

[B11] Bollig N. Brüstle A. Kellner K. Ackermann W. Abass E. Raifer H.. (2012). Transcription factor IRF4 determines germinal center formation through follicular T-helper cell differentiation. Proc. Natl. Acad. Sci. U S A 109, 8664–8669. doi: 10.1073/pnas.1205834109 22552227 PMC3365194

[B12] Cai Y. Yang Y. Chen X. Wu G. Zhang X. Liu Y.. (2016). Circulating ‘lncRNA OTTHUMT00000387022’ from monocytes as a novel biomarker for coronary artery disease. Cardiovasc. Res. 112, 714–724. doi: 10.1093/cvr/cvw022 26857419

[B13] Christensen S. M. Belew A. T. El-Sayed N. M. Tafuri W. L. Silveira F. T. Mosser D. M. (2019). Host and parasite responses in human diffuse cutaneous leishmaniasis caused by L. amazonensis. PloS Negl. Trop. Dis. 13, e0007152. doi: 10.1371/journal.pntd.0007152 30845223 PMC6405045

[B14] Costa D. L. Cardoso T. M. Queiroz A. Milanezi C. M. Bacellar O. Carvalho E. M.. (2015). Tr-1-like CD4+CD25-CD127-/lowFOXP3- cells are the main source of interleukin 10 in patients with cutaneous leishmaniasis due to Leishmania Braziliensis. J. Infect. Dis. 211, 708–718. doi: 10.1093/infdis/jiu406 25139022 PMC4402371

[B15] Dabitao D. Hedrich C. M. Wang F. Vacharathit V. Bream J. H. (2018). Cell-specific requirements for STAT proteins and type I IFN receptor signaling discretely regulate IL-24 and IL-10 expression in NK cells and macrophages. J. Immunol. 200, 2154–2164. doi: 10.4049/jimmunol.1701340 29436412 PMC5840025

[B16] Davis R. S. (2020). Roles for the FCRL6 immunoreceptor in tumor immunology. Front. Immunol. 11. doi: 10.3389/fimmu.2020.575175 PMC7591390 33162991

[B17] DiStefano J. K. (2018). “The Emerging Role of Long Noncoding RNAs in Human Disease,” in Disease Gene Identification: Methods and Protocols. Ed. DiStefano J. K. (Springer New York, New York, NY), 91–110. 10.1007/978-1-4939-7471-9_6 29423795

[B18] Dobin A. Davis C. A. Schlesinger F. Drenkow J. Zaleski C. Jha S.. (2013). STAR: ultrafast universal RNA-seq aligner. Bioinformatics. 29, 15–21. doi: 10.1093/bioinformatics/bts635 23104886 PMC3530905

[B19] Espitia C. M. Zhao W. Saldarriaga O. Osorio Y. Harrison L. M. Cappello M.. (2010). Duplex real-time reverse transcriptase PCR to determine cytokine mRNA expression in a hamster model of New World cutaneous leishmaniasis. BMC Immunol. 11, 31. doi: 10.1186/1471-2172-11-31 20569429 PMC2909172

[B20] Fabregat A. Sidiropoulos K. Viteri G. Forner O. Marin-Garcia P. Arnau V.. (2017). Reactome pathway analysis: a high-performance in-memory approach. BMC Bioinf. 18, 142. doi: 10.1186/s12859-017-1559-2 PMC5333408 28249561

[B21] Fakiola M. Singh O. P. Syn G. Singh T. Singh B. Chakravarty J.. (2019). Transcriptional blood signatures for active and amphotericin B treated visceral leishmaniasis in India. PloS Negl. Trop. Diseases 13, e0007673. doi: 10.1371/journal.pntd.0007673 PMC6713396 31419223

[B22] Fang K. Caixia H. Xiufen Z. Zijian G. Li L. (2020). Screening of a novel upregulated lncRNA, A2M-AS1, that promotes invasion and migration and signifies poor prognosis in breast cancer. BioMed. Res. Int. 2020, 9747826. doi: 10.1155/2020/9747826 32352014 PMC7171613

[B23] Farias Amorim C. ON F. Nguyen B. T. Nascimento M. T. Lago J. Lago A. S.. (2021). Localized skin inflammation during cutaneous leishmaniasis drives a chronic, systemic IFN-γ signature. PloS Negl. Trop. Dis. 15, e0009321. doi: 10.1371/journal.pntd.0009321 33793565 PMC8043375

[B24] Fernandes J. C. R. Goncalves A. N. A. Floeter-Winter L. M. Nakaya H. I. Muxel S. M. (2022). Comparative transcriptomic analysis of long noncoding RNAs in Leishmania-infected human macrophages. Front. Genet. 13, 1051568. doi: 10.3389/fgene.2022.1051568 36685903 PMC9845402

[B25] Finalet Ferreiro J. Rouhigharabaei L. Urbankova H. van der Krogt J.-A. Michaux L. Shetty S.. (2014). Integrative genomic and transcriptomic analysis identified candidate genes implicated in the pathogenesis of hepatosplenic T-cell lymphoma. PloS One 9, e102977. doi: 10.1371/journal.pone.0102977 25057852 PMC4109958

[B26] Finelli M. J. Oliver P. L. (2017). TLDc proteins: new players in the oxidative stress response and neurological disease. Mamm Genome 28, 395–406. doi: 10.1007/s00335-017-9706-7 28707022 PMC5614904

[B27] Galgamuwa L. S. Sumanasena B. Iddawela D. Wickramasinghe S. Yatawara L. (2019). Assessment of intralesional cytokine profile of cutaneous leishmaniasis caused by Leishmania donovani in Sri Lanka. BMC Microbiol. 19, 14. doi: 10.1186/s12866-018-1384-4 30642262 PMC6332851

[B28] Gardinassi L. G. Garcia G. R. Costa C. H. N. Costa Silva V. de Miranda Santos I. K. F. (2016). Blood transcriptional profiling reveals immunological signatures of distinct states of infection of humans with leishmania infantum. PloS Negl. Trop. Diseases 10, e0005123. doi: 10.1371/journal.pntd.0005123 PMC5102635 27828962

[B29] Jafarzadeh A. Nemati M. Chauhan P. Patidar A. Sarkar A. Sharifi I.. (2020). Interleukin-27 functional duality balances Leishmania infectivity and pathogenesis. Front. Immunol. 11, 1573. doi: 10.3389/fimmu.2020.01573 32849534 PMC7427467

[B30] Khatonier R. Khan A. M. Sarmah P. Ahmed G. U. (2018). Role of IL-21 in host pathogenesis in experimental visceral leishmaniasis. J. Parasit Dis. 42, 500–504. doi: 10.1007/s12639-018-1025-8 30538346 PMC6261154

[B31] Kima P. Soong L. (2013). Interferon gamma in leishmaniasis. Front. Immunol. 4. doi: 10.3389/fimmu.2013.00156 PMC3685816 23801993

[B32] Kohl M. Wiese S. Warscheid B. (2011). Cytoscape: software for visualization and analysis of biological networks. Data Min. proteomics: standards to Appl. 696, 291–303. doi: 0.1007/978-1-60761-987-1_18 10.1007/978-1-60761-987-1_18 21063955

[B33] Kolde R. (2012). “Pheatmap: pretty heatmaps,” in R package version, vol. 1. , 726.

[B34] Kong F. Saldarriaga O. A. Spratt H. Osorio E. Y. Travi B. L. Luxon B. A.. (2017). Transcriptional profiling in experimental visceral leishmaniasis reveals a broad splenic inflammatory environment that conditions macrophages toward a disease-promoting phenotype. PloS Pathog. 13, e1006165. doi: 10.1371/journal.ppat.1006165 28141856 PMC5283737

[B35] Kopp F. Mendell J. T. (2018). Functional classification and experimental dissection of long noncoding RNAs. Cell. 172, 393–407. doi: 10.1016/j.cell.2018.01.011 29373828 PMC5978744

[B36] Kresse A. Konermann C. Degrandi D. Beuter-Gunia C. Wuerthner J. Pfeffer K.. (2008). Analyses of murine GBP homology clusters based on *in silico*, *in vitro* and *in vivo* studies. BMC Genomics 9, 1–12. doi: 10.1186/1471-2164-9-158 18402675 PMC2387175

[B37] Langfelder P. Horvath S. (2008). WGCNA: an R package for weighted correlation network analysis. BMC Bioinf. 9, 1–13. doi: 10.1186/1471-2105-9-559 PMC2631488 19114008

[B38] Lecoeur H. Prina E. Gutiérrez-Sanchez M. Späth G. F. (2022). Going ballistic: Leishmania nuclear subversion of host cell plasticity. Trends Parasitol. 38, 205–216. doi: 10.1016/j.pt.2021.09.009 34666937

[B39] Lecoeur H. Prina E. Rosazza T. Kokou K. N'Diaye P. Aulner N.. (2020). Targeting macrophage histone H3 modification as a leishmania strategy to dampen the NF-κB/NLRP3-mediated inflammatory response. Cell Rep. 30, 1870–82.e4. doi: 10.1016/j.celrep.2020.01.030 32049017

[B40] Lin Y. Liu T. Cui T. Wang Z. Zhang Y. Tan P.. (2020). RNAInter in 2020: RNA interactome repository with increased coverage and annotation. Nucleic Acids Res. 48, D189–DD97. doi: 10.1093/nar/gkz804 31906603 PMC6943043

[B41] Liu S. Hur Y. H. Cai X. Cong Q. Yang Y. Xu C.. (2023). A tissue injury sensing and repair pathway distinct from host pathogen defense. Cell. 186, 2127–43.e22. doi: 10.1016/j.cell.2023.03.031 37098344 PMC10321318

[B42] Love M. I. Huber W. Anders S. (2014). Moderated estimation of fold change and dispersion for RNA-seq data with DESeq2. Genome Biol. 15, 1–21. doi: 10.1186/s13059-014-0550-8 PMC4302049 25516281

[B43] Lv X. Liu L. Li P. Yuan Y. Peng M. Jin H.. (2022). Constructing a novel signature based on immune-related lncRNA to improve prognosis prediction of cervical squamous cell carcinoma patients. Reprod. Sci. 29, 800–815. doi: 10.1007/s43032-022-00851-z 35075611

[B44] Lyu J. Zheng P. Qi Y. Huang G. (2023). LightGBM-lncLoc: A lightGBM-based computational predictor for recognizing long non-coding RNA subcellular localization. Mathematics. 11, 602. doi: 10.3390/math11030602

[B45] Maretti-Mira A. C. Bittner J. Oliveira-Neto M. P. Liu M. Kang D. Li H.. (2012). Transcriptome patterns from primary cutaneous Leishmania Braziliensis infections associate with eventual development of mucosal disease in humans. PloS Negl. Trop. Dis. 6, e1816. doi: 10.1371/journal.pntd.0001816 23029578 PMC3441406

[B46] Maruyama S. R. Fuzo C. A. Oliveira A. E. R. Rogerio L. A. Takamiya N. T. Pessenda G.. (2022). Insight into the long noncoding RNA and mRNA coexpression profile in the human blood transcriptome upon leishmania infantum infection. Front. Immunol. 13, 784463. doi: 10.3389/fimmu.2022.784463 35370994 PMC8965071

[B47] Masoudzadeh N. Mizbani A. Rafati S. (2020a). Transcriptomic profiling in Cutaneous Leishmaniasis patients. Expert Rev. Proteomics 17, 533–541. doi: 10.1080/14789450.2020.1812390 32886890

[B48] Masoudzadeh N. Östensson M. Persson J. Mashayekhi Goyonlo V. Agbajogu C. Taslimi Y.. (2020b). Molecular signatures of anthroponotic cutaneous leishmaniasis in the lesions of patients infected with Leishmania tropica. Sci. Rep. 10, 16198. doi: 10.1038/s41598-020-72671-7 33004861 PMC7529897

[B49] Mattick J. S. Amaral P. P. Carninci P. Carpenter S. Chang H. Y. Chen L.-L.. (2023). Long non-coding RNAs: definitions, functions, challenges and recommendations. Nat. Rev. Mol. Cell Biol. 24, 430–447. doi: 10.1038/s41580-022-00566-8 36596869 PMC10213152

[B50] Monteleone G. Pallone F. MacDonald T. T. (2008). Interleukin-21: a critical regulator of the balance between effector and regulatory T-cell responses. Trends Immunol. 29, 290–294. doi: 10.1016/j.it.2008.02.008 18440864

[B51] Panditrao G. Bhowmick R. Meena C. Sarkar R. R. (2022). Emerging landscape of molecular interaction networks:Opportunities, challenges and prospects. J. Biosci. 47, 24. doi: 10.1007/s12038-022-00253-y 36210749 PMC9018971

[B52] Porta C. Rimoldi M. Raes G. Brys L. Ghezzi P. Di Liberto D.. (2009). Tolerance and M2 (alternative) macrophage polarization are related processes orchestrated by p50 nuclear factor κB. Proc. Natl. Acad. Sci. 106, 14978–14983. doi: 10.1073/pnas.0809784106 19706447 PMC2736429

[B53] Qiu X. Shi Q. Zhang X. Shi X. Jiang H. Qin S. (2022). LncRNA A2M-AS1 promotes ferroptosis in pancreatic cancer via interacting with PCBP3. Mol. Cancer Res. 20, 1636–1645. doi: 10.1158/1541-7786.MCR-22-0024 35920801

[B54] Quirino G. F. Nascimento M. S. Davoli-Ferreira M. Sacramento L. A. Lima M. H. Almeida R. P.. (2016). Interleukin-27 (IL-27) mediates susceptibility to visceral leishmaniasis by suppressing the IL-17–neutrophil response. Infection immunity 84, 2289–2298. doi: 10.1128/IAI.00283-16 27245409 PMC4962641

[B55] Rosas L. E. Satoskar A. A. Roth K. M. Keiser T. L. Barbi J. Hunter C.. (2006). Interleukin-27R (WSX-1/T-cell cytokine receptor) gene-deficient mice display enhanced resistance to leishmania donovani infection but develop severe liver immunopathology. Am. J. pathology 168, 158–169. doi: 10.2353/ajpath.2006.050013 PMC1592683 16400019

[B56] Ruiz-Postigo J. A. Jain S. Mikhailov A. Maia-Elkhoury A. N. Valadas S. Warusavithana S.. (2021). Global leishmaniasis surveillance: 2019-2020, a baseline for the 2030 roadmap/Surveillance mondiale de la leishmaniose: 2019-2020, une periode de reference pour la feuille de route a l'horizon 2030. Weekly epidemiological Rec. 96, 401–420.

[B57] Sa S. M. Valdez P. A. Wu J. Jung K. Zhong F. Hall L.. (2007). The effects of IL-20 subfamily cytokines on reconstituted human epidermis suggest potential roles in cutaneous innate defense and pathogenic adaptive immunity in psoriasis. J. Immunol. 178, 2229–2240. doi: 10.4049/jimmunol.178.4.2229 17277128

[B58] Schoggins J. W. (2019). Interferon-stimulated genes: what do they all do? Annu. Rev. Virol. 6, 567–584. doi: 10.1146/annurev-virology-092818-015756 31283436

[B59] Scott P. Novais F. O. (2016). Cutaneous leishmaniasis: immune responses in protection and pathogenesis. Nat. Rev. Immunol. 16, 581–592. doi: 10.1038/nri.2016.72 27424773

[B60] Shi J. Ding W. Lu H. (2020). Identification of long non-coding RNA SNHG family as promising prognostic biomarkers in acute myeloid leukemia. OncoTargets Ther. 13, 8441–8450. doi: 10.2147/OTT.S265853 PMC7457734 32922034

[B61] Statello L. Guo C. J. Chen L. L. Huarte M. (2021). Gene regulation by long non-coding RNAs and its biological functions. Nat. Rev. Mol. Cell Biol. 22, 96–118. doi: 10.1038/s41580-020-00315-9 33353982 PMC7754182

[B62] Taslimi Y. Sadeghipour P. Habibzadeh S. Mashayekhi V. Mortazavi H. Müller I.. (2017). A novel non-invasive diagnostic sampling technique for cutaneous leishmaniasis. PloS Negl. Trop. Diseases 11, e0005750. doi: 10.1371/journal.pntd.0005750 PMC5526608 28704463

[B63] Team R Team RC (2018). R Foundation for Statistical Computing (Vienna: R-project org).

[B64] Tian S. Tang M. Li J. Wang C. Liu W. (2020). Identification of long non-coding RNA signatures for squamous cell carcinomas and adenocarcinomas. Aging (Albany NY) 13, 2459–2479. doi: 10.18632/aging.202278 33318305 PMC7880362

[B65] Tretina K. Park E. S. Maminska A. MacMicking J. D. (2019). Interferon-induced guanylate-binding proteins: Guardians of host defense in health and disease. J. Exp. Med. 216, 482–500. doi: 10.1084/jem.20182031 30755454 PMC6400534

[B66] Vandooren J. Itoh Y. (2021). Alpha-2-macroglobulin in inflammation, immunity and infections. Front. Immunol. 12, 803244. doi: 10.3389/fimmu.2021.803244 34970276 PMC8712716

[B67] Villarino A. Hibbert L. Lieberman L. Wilson E. Mak T. Yoshida H.. (2003). The IL-27R (WSX-1) is required to suppress T cell hyperactivity during infection. Immunity. 19, 645–655. doi: 10.1016/S1074-7613(03)00300-5 14614852

[B68] Wang K. C. Chang H. Y. (2011). Molecular mechanisms of long noncoding RNAs. Mol. Cell. 43, 904–914. doi: 10.1016/j.molcel.2011.08.018 21925379 PMC3199020

[B69] Weighill D. Ben Guebila M. Glass K. Platig J. Yeh J. J. Quackenbush J. (2021). Gene targeting in disease networks. Front. Genet. 12. doi: 10.3389/fgene.2021.649942 PMC8103030 33968133

[B70] Wertheimer E. Gutierrez-Uzquiza A. Rosemblit C. Lopez-Haber C. Sosa M. S. Kazanietz M. G. (2012). Rac signaling in breast cancer: a tale of GEFs and GAPs. Cell. signalling 24, 353–362. doi: 10.1016/j.cellsig.2011.08.011 21893191 PMC3312797

[B71] Ye L. Jin W. (2021). Identification of lncRNA-associated competing endogenous RNA networks for occurrence and prognosis of gastric carcinoma. J. Clin. Lab. Anal. 35, e24028. doi: 10.1002/jcla.v35.12 34704289 PMC8649378

[B72] Zhang S. O'Regan R. Xu W. (2020). The emerging role of mediator complex subunit 12 in tumorigenesis and response to chemotherapeutics. Cancer. 126, 939–948. doi: 10.1002/cncr.v126.5 31869450 PMC7021580

[B73] Zhang X. Wang W. Zhu W. Dong J. Cheng Y. Yin Z.. (2019). Mechanisms and functions of long non-coding RNAs at multiple regulatory levels. Int. J. Mol. Sci. 20, 5573. doi: 10.3390/ijms20225573 31717266 PMC6888083

[B74] Zheng H. Wang G. Wang Y. Liu J. Ma G. Du J. (2023). Systematic analysis reveals a pan-cancer SNHG family signature predicting prognosis and immunotherapy response. iScience. 26, 108055. doi: 10.1016/j.isci.2023.108055 37854704 PMC10579433

[B75] Zhou W. Liu T. Saren G. Liao L. Fang W. Zhao H. (2019). Comprehensive analysis of differentially expressed long non-coding RNAs in non-small cell lung cancer. Oncol. Lett. 18, 1145–1156. doi: 10.3892/ol.2019.10414 31423174 PMC6607379

[B76] Zimta A.-A. Tigu A. B. Braicu C. Stefan C. Ionescu C. Berindan-Neagoe I. (2020). An emerging class of long non-coding RNA with oncogenic role arises from the snoRNA host genes. Front. Oncol. 10. doi: 10.3389/fonc.2020.00389 PMC7154078 32318335

